# Client perspectives of internet-based treatment for depression in Arabic-speaking countries

**DOI:** 10.1186/s40359-026-04463-z

**Published:** 2026-04-13

**Authors:** Rayan El-Haj-Mohamad, Laura Nohr, Nadine Stammel, Zaid Salloum, Maya Böhm, Yuriy Nesterko, Birgit Wagner, Maria Böttche, Christine Knaevelsrud

**Affiliations:** 1 Clinical Psychological Intervention, Department of Education and Psychology, Freie Universität Berlin, Berlin, Germany; 2 Center ÜBERLEBEN, Berlin, Germany; 3 Department of Medical Psychology and Medical Sociology, University of Leipzig, Leipzig, Germany; 4 Clinical Psychology and Psychotherapy, Medical School Berlin, Berlin, Germany; 5 German Center for Mental Health (DZPG), Partner Site Berlin-Potsdam, Berlin-Potsdam, Germany

**Keywords:** Interpersonal psychotherapy, Cognitive behavioral therapy, Online therapy, E-mental health, User experience, MENA region

## Abstract

**Background:**

Mental health care faces significant challenges, particularly in Arabic-speaking countries, where only a fraction of individuals with depression receive treatment. While internet-based interventions (IBIs) have shown promising results, how clients experience IBIs is not well known, highlighting the need for a deeper understanding of client experiences.

**Objective:**

To better understand the impact of IBIs, qualitative interviews were conducted with participants from Arabic-speaking countries regarding perceived changes, causes of changes, and hindering and helpful aspects of IBIs.

**Method:**

In total, 93 participants (68% female; M_age = 27.45) from various Arabic-speaking countries who completed either an internet-based cognitive (*n* = 49) or an internet-based interpersonal (*n* = 44) treatment for depression were interviewed via structured change interviews to understand perceived positive and negative changes after treatment as well as perceived causes of changes, as well as helpful and hindering aspects. The interviews were analyzed using qualitative content analysis.

**Results:**

Most of the participants reported positive changes after treatment, which were categorized into the five following domains: Coping strategies, Personal Growth, Interpersonal Changes, Symptomatic Changes and Increase in Activity and Energy. Causes of changes were attributed mainly to the Program Structure, Personal Factors, Working Alliance, and External Reasons. However, negative changes were also reported and categorized into Short-term and Persistent Negative Changes. Hindering aspects were related to the Program Structure and Technical Difficulties, Standardization and External Reasons. Helpful aspects were related to the Program Structure.

**Conclusion:**

Despite some hindering aspects and negative changes, the IBIs predominantly induced positive changes across multiple life domains, with the Program Structure being identified as both the main cause of positive effects and an obstacle, which indicates individual-dependent differences.

**Supplementary Information:**

The online version contains supplementary material available at 10.1186/s40359-026-04463-z.

## Introduction

Depression represents one of the leading causes of disability worldwide, with an increasing trend over time [61]. This pattern is particularly evident in Arabic-speaking countries, where Moradinazar et al. [40] reported a rising rate of affective disorders. Despite the high prevalence of depression, a recent meta-analysis and systematic review indicated that only 34.8% of individuals with depression receive any treatment globally, with particularly lower treatment rates in low-income countries (16.8%) and significantly lower rates in Arabic-speaking countries (compared with Europe; [38]). To address these treatment gaps, innovative approaches to treatment delivery have been developed. Internet-based interventions (IBIs) are promising approaches and have become increasingly important for addressing global mental health challenges, especially in times of crises (e.g. COVID-19 pandemic; [36, 42, 43, 62]. The efficacy of IBI is well documented in research. Multiple studies have demonstrated moderate to strong effects for IBIs in treating depression, showing comparable results to traditional face-to-face settings [15, 26, 41, 57]. Cognitive Behavioral Therapy (CBT) are primarily used in IBIs [41]. CBT is based on the understanding that psychological distress stems partly from maladaptive thinking patterns and learned behaviors, which can be transformed through the development of more adaptive coping strategies, with a primary focus on identifying and modifying dysfunctional cognitive schemas and their interaction with emotional and behavioral components ([5]). Interpersonal psychotherapy (IPT) includes CBT components and emphasizes the importance of interpersonal relationships and social interactions to improve social competencies and actively shape functional relationship patterns [29]. The latter approach might be particularly relevant in Arabic-speaking countries, where community values play a central role in daily life [23, 24]. While most findings come from Western, educated, industrialized, rich, and democratic samples, the first study demonstrated the efficacy of these methods in Arabic-speaking samples [28]. A recent study revealed that internet-based CBT (iCBT) and internet-based IPT (iIPT) are equally effective in treating depression among Arabic-speaking individuals. Both treatments resulted in significant symptom reduction compared with a waitlist control group [16].

While researchers have analyzed various predictors and moderators of treatment outcomes, only baseline depression severity has been identified as a consistently significant influence [55]. Factors such as gender, education, and relationship status have not shown significant predictive or moderating effects [13].These inconsistent findings could indicate that quantitative approaches might be insufficient for predicting but also understanding treatment success and adherence. In addition, qualitative research might be valuable for understanding patients’ subjective experiences in depth. A substantial portion of therapeutic success is attributed to client change processes, with estimates ranging from 40% [3] to 87% [59] in face-to-face treatment. Despite this significant contribution, several researchers have highlighted the neglected factor of the client perspective in psychotherapy research [8]. The neglected focus on clients' own subjective experiences and perspectives is described by Levitt et al. [34] as the 'forgotten client' in psychotherapy research. While McPherson et al. [37] emphasized that treatment guidelines often fail to include qualitative research on patient experiences, this perspective is crucial for improving treatment effectiveness.

The insights gained from qualitative approaches have been substantial. A recent meta-analytical qualitative review by Ladmanová et al. [32] including 17 articles identified twelve helpful categories in traditional one-by-one treatment methods, including gaining new perspectives on the self (e.g., understanding one’s own behavior better), experiencing relief (e.g., feeling more relaxed), and feeling understood (e.g., feeling heard, accepted). This study also revealed eight hindering categories, such as difficulties in disclosing (e.g., sharing feelings of anger toward family), feeling emotionally overwhelmed (e.g., dealing with specific thoughts), having difficulties accomplishing tasks (e.g., feeling unprepared to complete tasks) or lacking therapeutic guidance (e.g., needing more instructions). In their qualitative analysis of outcome domains for depression, which included *N* = 3003 participants (patients, caregivers and healthcare professionals), Chevance et al. [11] identified, in addition to domains of symptom reduction, domains of improvement in functioning, such as social functioning or elementary functioning, as well. McPherson et al. [37] conducted a comprehensive qualitative meta-analysis (*k* = 38) examining and comparing different treatment methods, including IBIs. Their analysis revealed important common factors across psychological therapies (e.g., gaining new insights to reframe emotions). They also highlighted that remote therapies could enhance accessibility and reduce stigma but often face limitations in terms of client satisfaction due to reduced interaction with therapists. Their review emphasized that psychological models and therapeutic techniques focusing on individual problems may have limited relevance to individuals' everyday lives, particularly in addressing immediate family or social problems that may trigger or maintain depressive experiences.

While qualitative studies examining participants' experiences in IBIs are generally scarce, those that do exist predominantly focus on samples from Western countries [32, 44], leaving a significant gap in understanding the experiences of participants from underrepresented groups. This gap is particularly noteworthy given the findings of McPherson et al. [37], who focused on working with specific ethnic groups or economically disadvantaged populations. Research has shown that therapeutic approaches addressing practical issues beyond established frameworks are more successful in enhancing therapeutic relationships and improving outcomes. A recent attempt to bridge this gap was made by Lindegaard et al. [35], who investigated the experiences of Arabic-speaking participants using iCBT for depression and anxiety in Sweden. Their study identified five key themes, including “the importance of being seen”. This theme describes participants’ experiences of trusting relationships with their therapists, while some wished for more voice contact and "new ways of knowing", which described an increased understanding of their situation and problems. However, these findings may not be fully translatable to participants in Arabic-speaking countries, where healthcare systems and cultural contexts differ substantially from those in Sweden.

Thus, a critical knowledge gap remains: while emerging evidence demonstrates that IBIs can effectively reduce depressive symptoms in Arabic-speaking populations [16, 28], we lack understanding of how participants experience the change process, what they perceive as helpful or hindering. Given that therapeutic approaches addressing issues beyond established frameworks have shown greater success [37], understanding the subjective experiences of Arabic-speaking participants receiving IBIs is essential for optimizing intervention design and implementation.

The present study addresses this gap by exploring the experiences of Arabic-speaking participants who completed internet-based treatment for depression (iCBT or iIPT). Specifically, we aimed to answer the following research questions:

1. What changes in well-being and daily life do participants report following IBI participation, including both positive and negative changes?
2. What aspects of the intervention do participants perceive as helpful or hindering?
3. To what factors do participants attribute the changes they experienced?

## Method

The present qualitative study is part of the project “Ilajanafsy” (Arabic for psychotherapy), which offers psychological treatment for people in Arabic-speaking countries suffering from depression.

### Treatment

The *Ilajnafsy* program was developed on the basis of the Interapy protocol [33]. The iCBT approach was developed on the basis of Beck's model ([4]), and the iIPT approach was developed on the basis of components of the interpersonal therapeutic approach [29]. Both iCBT and iIPT consisted of two 45-min weekly structured writing tasks over a period of five weeks. Trained counselors responded individually to participants' written work within 48 h. At the beginning of the treatment, the participants were informed about the concept and procedure of internet-based treatment.

#### Internet-based cognitive behavioral treatment

The initial stage, known as behavioral analysis, involves two writing tasks and focuses on supporting participants in understanding their depressive symptoms. The next stage, behavioral activation, also includes two writing tasks and aims to incorporate positive activities into the participants' daily routines. In the third stage, cognitive restructuring, three writing tasks are utilized to replace unhelpful thoughts with more adaptive thoughts and to develop new skills, such as setting boundaries. The final stage for relapse prevention aims at reducing the risk of relapse by creating an emergency plan.

##### Internet-based interpersonal psychotherapy

The initial stage, communication analysis, involves two writing tasks and focuses on evaluating the participant's communication skills. The second stage aims at clarifying and coping with interpersonal conflicts and includes three writing tasks that support participants in understanding individual role conflicts in their relationships. The third stage addresses withdrawal behavior and isolation, consists of three writing tasks and aims to help participants understand the effects of isolation and develop coping strategies. The final relapse prevention stage aims to minimize the risk of relapse by creating an emergency plan.

### Participants

Arabic-speaking participants who fulfilled the inclusion criteria (provided informed consent to study policies, age ≥ 18 years, Arabic language proficiency, and at least mild depressive symptoms indicated by a BDI score > 13), did not meet the exclusion criteria (current suicidal tendencies, severe depression indicated by a BDI score > 44, current psychosis, substance abuse, ongoing psychotherapy, or unstable psychopharmacological medication) and met diagnostic criteria for depression according to the Structured Clinical Interview for DSM-5 Disorders (SCID-5-CV; [20]) were randomly assigned to a guided internet-based intervention [16]. Full details of the recruitment procedure are reported in [16]. After the treatment, the participants were contacted online for a voice-over-IP interview. Participants who regularly completed the treatment for depression (iCBT or iIPT) and agreed to participate in the interview were eligible for the current study. Participation in the study was voluntary, and no compensation or incentives were provided.

### Instruments

The following quantitative measures were used to characterize the sample at pre- and post-treatment; detailed descriptions are provided in the parent RCT [16]. Depressive symptoms were assessed using an Arabic version of the Patient Health Questionnaire-9 (PHQ-9, [30], Arabic validation: [2, 52], α = 0.78). Anxiety symptoms were measured with an Arabic version of the Generalized Anxiety Disorder-7 scale (GAD-7; [56], Arabic validation: [52], α = 0.83). Quality of life was assessed using the EUROHIS-QoL-8 [54], international cross-cultural validity has been demonstrated [45, 54], α = 0.75). Perceived social support was measured with a validated Arabic version of the Multidimensional Scale of Perceived Social Support (MSPSS; [63], Arabic validation: [19], Merhi et al. [39]; α = 0.90). All internal consistencies are reported from the parent study sample [16].

### Interview

The structured Change Interview [17, 18] was used to explore the clients’ perspectives and perceived experiences of changes since treatment started, the perceived causes of changes, and the impact of those changes, including helpful and hindering aspects of treatment. The interview consists of six to eight questions. For example, “What changes, if any, have you noticed in yourself since therapy started?”,“In general, what do you think has caused these various changes?”. The interview was translated from English to Arabic via the back-to-forth translation approach by independent native Arabic speakers.

### Procedure

Following completion of the treatment and post-assessment, participants were contacted via the messaging system of the program to schedule the feedback interviews (i.e., Change Interview; [17, 18]). If participants responded, interviews proceeded as scheduled. When no response was given, the interviewer made one active telephone contact attempt. The feedback interviews remained accessible for 21 days before being marked as "missed." Prior to the interview, participants provided informed consent for their participation in the qualitative study.

Interviews were conducted in Arabic via voice-over-IP by Arabic-speaking team interviewers. Counselors did not interview their own clients to allow participants to answer as openly as possible. Interviewee responses were noted by the interviewers during the interviews. No audio recordings were made; therefore, exact interview durations are not available. Interviewer notes were consolidated into a standardized format through the *Ilajnafsy* program to ensure consistency for subsequent qualitative analysis.

### Data analysis

To illustrate the descriptive characteristics of the sample who agreed to participate in an interview, R version 2023.03.0 + 386 was used. Furthermore, analyses comparing sociodemographic and mental health characteristics between participants who agreed to participate in an interview and participants who did not agree to participate in an interview were conducted via Welch tests, chi-square tests, or t tests. The interviews were analyzed in the original language via the deductive-inductive qualitative content approach [31]. Each piece of relevant information was provided with a code regardless of whether it was a single word or a longer sentence or paragraph. The codes were summarized quantitatively. Coding was realized on a semantic level and descriptively. The participants’ responses were interpreted as their lived experiences. No additional critical analysis was performed. Comparably, themes were defined as patterns in the data summarizing important information on a semantic level.

To familiarize themselves with the data, the first author (RE) read the participants’ answers several times before starting to code the data. RE then developed a coding system inductively using 15% of the data, as suggested by Kuckartz [31]. Second, the developed coding system was discussed with LN and NS. Third, all the interviews were analyzed and coded according to the coding system, with the possibility of adding new categories when new themes were mentioned. As a quality criterion, 15% of the data were coded twice by a ZS. To mitigate biases, the two raters were blinded to the treatment group and individual treatment outcome. Discrepancies were discussed between the two raters. The degree of agreement between the raters was high, at 90%. Additional quality criteria were fulfilled by focusing on maintaining thematic distinctiveness: we aimed for internal homogeneity within themes (ensuring that all data within a theme cohered meaningfully) while establishing sufficient heterogeneity between themes (ensuring clear distinguishability between different themes). This was achieved by applying clear and distinctive definitions for categories, which were illustrated via examples (Kuckartz et al., 2018; Supplement 1). Furthermore, an iterative process of reviewing and refining themes, checking that coded extracts formed coherent patterns within themes while maintaining clear boundaries between different themes, ensured the fulfillment of the quality criteria. The chosen quotations for that study were translated as closely as possible to the original wording by RE. Linguistic inaccuracies or grammatical errors have been retained to preserve the authenticity of the statements. The qualitative analysis was performed via MAXQDA 24 for Windows.

## Results

Between March 2021 and May 2024, 525 participants completed the treatments offered via the *Ilajnafsy* platform, of whom 93 consented to participate in posttreatment interviews, comprising 49 participants who received iCBT and 44 participants who received iIPT. As only 17.7% of all contacted participants agreed to participate in an interview, a comparison of sociodemographic and clinical characteristics was conducted. Compared with nonexposed participants, interviewed participants presented significantly lower rates of posttreatment depressive and anxiety symptoms (Table 1). Most of the participants were female, highly educated, single, and lived in a town. The majority reported being from Egypt (*n* = 38), Saudi Arabia (*n* = 12), Syria (*n* = 10), Iraq (*n* = 8), Algeria (*n* = 6), Jordan (*n* = 4) and the United Arabic Emirates (*n* = 3). Other reported countries of origin were Ethiopia, Lebanon, Malaysia, Palestine, Turkey, Ukraine, and Yemen. For further characteristics, see Table 2.

**Table 1:** Comparison of non-interviewed and interviewed participants*

		**Non-interviewed (*n* = 432)**	**Interviewed (*n* = 93)**	**Chi-square/t test**	**p**
Age	*M* (*SD*)	26.63 (8.62)	27.45 (8.22)	.84	.40
Female	*n* (%)	295 (68.29)	68 (73.12)	.84	.36
High education^a^	*n* (%)	411 (95.14)	88 (94.62)	.043	.835
Town^b^	*n* (%)	383 (88.66)	79 (84.95)	.998	.318
iCBT^c^	*n (%)*	235 (54.90)	49 (52.69)	.09	.76
Family social support^d^ T0	*M* (*SD*)	3.38 (1.73)	3.13 (1.70)	−1.25	.21
Friends social support^d^ T0	*M* (*SD*)	3.19 (1.79)	3.28 (1.85)	.44	.66
Significant other social support^d^ T0	*M* (*SD*)	3.63 (1.89)	3.65 (1.75)	.09	.93
Depressive symptom severity (PHQ-9) T0	*M* (*SD*)	16.84 (5.19)	16.56 (5.03)	-.48	.63
Anxiety symptom severity (GAD-7) T0	*M* (*SD*)	13.93 (4.80)	13.94 (4.76)	.02	.99
Quality of life (EUROHIS-QOL-8) T0	*M* (*SD*)	13.30 (4.92)	12.60 (4.89)	−1.24	.99
Family social support^d^ T1	*M* (*SD*)	4.16 (1.82)	4.16 (1.82)	-.012	.99
Friends social support^d^ T1	*M* (*SD*)	4.90 (1.63)	3.87 (1.74)	−1.02	.31
Significant other social support^d^ T1	*M* (*SD*)	4.54 (1.91)	4.78 (1.75)	−1.08	.28
Depressive symptom severity (PHQ-9) T1	*M* (*SD*)	7.4 (5.77)	6.04 (4.79)	−2.11	**.035**
Anxiety symptom severity (GAD-7) T1	*M* (*SD*)	6.90 (5.38)	5.55 (4.26)	2.63	**.009**
Quality of life (EUROHIS-QOL-8) T1	*M* (*SD*)	19.26 (6.03)	19.98 (5.78)	−1.03	.30

significant differences (*p* < .05) are printed in bold

*M* Mean, *SD* Standard Deviation, *T0* Before treatment (baseline), *T1* Post treatment, *PHQ-9* Patient Health Questionnaire-9, *GAD-7* Generalized Anxiety Disorder Scale-7, *EUROHIS-QOL*  EUROHIS Quality of Life 8-item index

^a^Education was dichotomously coded as high (high school or university/college diploma) or low education (no or intermediate school diploma)

^b^Kind of residence was dichotomy coded in town (metropolitan city, small town, suburb) or village (village or single farmstead)

^c^iParticipants were treated with iCBT = internet-based cognitive behavioral treatment or iIPT = internet-based interpersonal treatment

^d^Perceived social support was measured via the multidimensional scale of perceived social support

*Psychometric characteristics of the used instrument are described inEl-Haj-Mohamad et al.[16]

**Table 2:** Sociodemographic, and clinical characteristics of the total sample and subsamples in each condition*

		**Total (*N* = 93)**	**iIPT(*n* = 44)**	**iCBT (*n* = 49)**	
Age	*M* (*SD*)	27.45 (8.22)	28.39 (7.68)	26.61 (8.67)	
Female	*n* (%)	68 (73.1)	35 (79.5)	33 (67.3)	
High education^a^	*n* (%)	88 (94.6)	43 (97.7)	45 (91.8)	
Town^b^	*n* (%)	79 (84.9)	36 (81.8)	43 (87.8)	
Family social support^d^ T0	*M* (*SD*)	3.13 (1.7)	3.23 (1.58)		3.04 (1.82)
Friends social support^d^ T0	*M* (*SD*)	3.28 (1.86)	3.52 (1.92)		3.07 (1.79)
Significant other social support^d^ T0	*M* (*SD*)	3.65 (1.75)	3.79 (1.74)		3.53 (1.77)
Depressive symptom severity (PHQ-9) T0	*M* (*SD*)	16.56 (5.03)	16.52 (4.89)		16.59 (5.2)
Anxiety symptom severity (GAD-7) T0	*M* (*SD*)	13.94 (4.76)	13.23 (4.75)		14.57 (4.73)
Quality of life (EUROHIS-QOL-8) T0	*M* (*SD*)	12.6 (4.89)	12.8 (4.62)		12.43 (5.16)
Family social support^d^ T1	*M* (*SD*)	4.16 (1.8)	4.30 (1.70)		4.04 (1.90)
Friends social support^d^ T1	*M* (*SD*)	4.35 (1.76)	4.90 (1.63)		3.87 (1.74)
Significant other social support^d^ T1	*M* (*SD*)	4.78 (1.75)	5.24 (1.42)		4.37 (1.91)
Depressive symptom severity (PHQ-9) T1	*M* (*SD*)	6.04 (4.79)	5.55 (4.65)		6.49 (4.9)
Anxiety symptom severity (GAD-7) T1	*M* (*SD*)	5.55 (4.62)	4.48 (4.15)		6.51 (4.17)
Quality of life (EUROHIS-QOL-8) T1	*M* (*SD*)	19.98 (5.78)	20.52 (5.42)		19.49 (6.10)

*M* = Mean, *SD* Standard Deviation, *T0* Before treatment (baseline), *T1* Post treatment, *PHQ-9* Patient Health Questionnaire-9, *GAD-7* Generalized Anxiety Disorder Scale-7, *EUROHIS-QOL* EUROHIS Quality of Life 8-item index

^a^Education was dichotomously coded as high (high school or university/college diploma) or low education (no or intermediate school diploma)

^b^Kind of residence was dichotomy coded in town (metropolitan city, small town, suburb) or village (village or single farmstead)

^c^iParticipants were treated with iCBT = internet-based cognitive behavioral treatment or iIPT = internet-based interpersonal treatment

^d^Perceived social support was measured via the multidimensional scale of perceived social support

^*^Psychometric Characteristics of the used instrument are described inEl-Haj-Mohamad et al. [16]

**Fig. 1:**
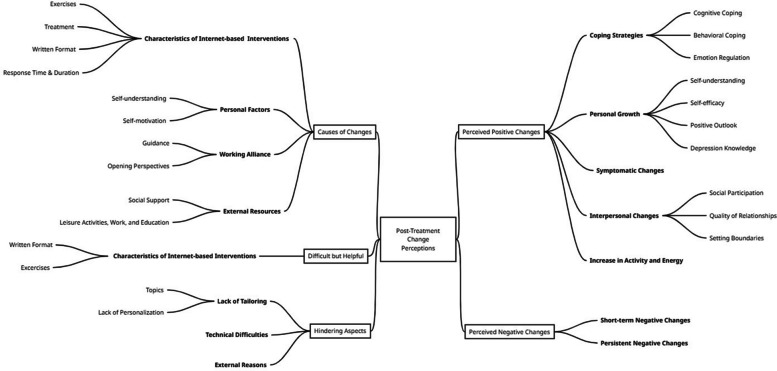
Overview of identified themes and subthemes. Notes: Themes are printed in bold, subthemes are printed normal

The following results are structured as follows. First, the subjective (positive and negative) changes experienced by the clients are described. Second, the causes of changes from the client's perspective are presented. Third, hindering, and fourth, difficult but helpful aspects are shown. For all parts, percentages for categories are described separately for iCBT and iIPT participants. The following percentages refer to the respective individual subject areas and do not necessarily add up to 100%, as the categories were evaluated independently of each other. An overview of all identified themes and subthemes is given in Fig. 1. Original quotes in Arabic are presented in Supplement 2.

### Perceived changes

In general, positive and negative results as well as no changes were reported. Positive changes were coded in the following five categories: Symptomatic changes, coping strategies, personal growth, interpersonal changes, and Increase in Activity and Energy. Negative changes were coded as *Short-term Negative Changes* and *Persistent Negative Changes.* Among iCBT clients, 8.2% (*n* = 4) and among iIPT clients, 11.4% (*n* = 5) reported not having recognized any changes (i.e., neither positive nor negative changes). Negative changes after treatment were reported by 10.2% of the iCBT participants and 13.6% of the iIPT participants. The three most often reported positive changes after iCBT were coping strategies, personal growth, and symptomatic changes, and after iIPT, they were personal growth, interpersonal changes, and coping strategies. The frequencies of subjectively experienced changes are shown in Table 3 separately for the treatment conditions.

**Table 3:** Positive changes perceived by participants separated by treatment group

Main category	Subcategory	iCBT (*n* = 49)	iIPT (*n* = 44)
**Symptomatic Changes**		**38.8% (19)**	**29.5% (13)**
**Coping Strategies**		**59.1% (29)**	**36.3% (16)**
	Cognitive coping	44.8% (22)	20.4% (9)
	Behavioral coping	10.2% (5)	6.8% (3)
	Emotion regulation	4.1% (2)	9.1% (4)
**Personal Growth**		**38.8% (19)**	**45.4% (20)**
	Self-understanding	14.3% (7)	15.9% (7)
	Self-efficacy	12.2% (6)	18.2% (8)
	Positive outlook	8.2% (4)	9.1% (4)
	Depression knowledge	4.1% (2)	2.3% (1)
**Interpersonal Changes**		**26.5% (13)**	**40.9% (18)**
	Social participation	12.2% (6)	18.2% (8)
	Quality of relationships	12.2% (6)	11.4% (5)
	Setting boundaries	4.1% (2)	11.4% (5)
**Increased Activity and Energy**		**26.5% (13)**	**20.5% (9)**

*iCBT* internet-based cognitive behavioral treatment, *iIPT* internet-based interpersonal treatment; multiple categories could be coded for each participant. The numbers in bold represent the main categories

#### Positive changes—symptomatic changes

The iCBT (38.8%, *n* = 19) and iIPT (29.5%; *n* = 13) participants reported a positive impact of the treatment on different symptoms. The participants reported shifts in core depressive symptomatology and mood state, as shown in the following quote: "*I have noticed a change for the better. I am turning from a depressed person to a happy person. I am back to normal now*." (iCBT16*)*. The severity of depressive symptoms was also reported to be reduced, as shown in the following quote from one participant: "*Depressive attacks have become less severe*" (iCBT1). Symptom improvements encompassed multiple somatic domains, as described below: "*Symptoms improved greatly, both sleep and appetite*" (iCBT32). A reduction in specific physical complaints was also reported, e.g., "*The physical symptoms of back pain and stomach pain have improved greatly*" (iIPT44). The comprehensive nature of these improvements is captured in the following quote: "*I was secretive, depressed, sad, and had little energy, and all of the negative thoughts have changed*" (iIPT9), indicating simultaneous improvements across multiple symptom domains.

#### Positive changes – coping strategies

The next identified theme, Coping Strategies focuses on actively used strategies to address symptoms or situations. We distinguished between emotion regulation, cognitive coping, behavioral coping, and depression knowledge. While the first is described as active control and management of emotions, the second is defined as strategies and problem-solving approaches on a cognitive level. Behavioral coping is described as specific behavioral actions to cope with symptoms. Reported changes in coping strategies after internet-based treatment were reported by 59.1% (*n* = 29) of iCBT and 36.3% (*n* = 16) of iIPT clients.

##### Cognitive coping

The most mentioned subcategory was cognitive coping, in which 44.8% (*n* = 22) of iCBT and 20.4% (*n* = 9) of iIPT participants reported having a new understanding of their symptoms and problems after treatment as well as a more aware understanding of the impact of thoughts on wellbeing and self. The theme is described through an active change of thoughts to be more helpful. The participants reported adopting more sophisticated analytical approaches to situations: "*I think about situations, analyze them, look for the negatives and positives*" (iCBT47). This was accompanied by the development of cognitive reframing abilities, as demonstrated by participant iCBT27's statement about actively seeking positive aspects within negative situations: "*I started looking for something positive in that negative situation to balance it out.*" (iCBT27). Furthermore, participants reported being able to influence thoughts in a more helpful way: "*I began to control negative thoughts*" (iCBT44) as well as trying to find alternatives: “*I started to think before I did anything, and not to give in to my negative thoughts and how to create alternatives to the things* “ (iIPT29*).*

##### Behavioral coping

The participants reported using different active strategies at the behavioral level to address their symptoms (10.2%, *n* = 5 iCBT; 6.8%, *n* = 3 iIPT). This underlines how the treatment methods could still be used in daily life even after finishing treatment. The following quote represents the category: "*I learned that when I was upset to write down the things that bothered me, and I also tried to solve negative situations. I had a change in my thoughts and behavior, and I became aware that when I was upset, I did breathing exercises, and I went out and walked and did something about my day*" (iCBT11).

##### Emotional regulation

The iCBT (4.1%, *n* = 2) and iIPT (9.1%, *n* = 4) participants reported better emotional regulation with multiple facets of emotional processing, including control, expression, awareness, and response modulation. These participants reported having better control over their emotions: "*I started to be able to control my feelings*" (iIPT36), and being able to recognize and express them in a better way: "*I began to express more feelings without accumulations*" (iIPT25*ت*). Furthermore, participants realized a decrease in the intensity of their emotional reactions: "*my emotional reactions have reduced compared to before*" (iCBT24).

#### Positive changes—personal growth

Personal Growth emerged as a distinct category representing changes in participants' self-perception, understanding, and outlook. While coping strategies represent situational skills and techniques that participants actively apply in challenging moments, personal growth reflects deeper, more enduring transformations in how participants see themselves, their condition, and their future. The participants in the iCBT (26.5%, *n* = 19) and iIPT (34.1%, *n* = 20) groups reported greater self-awareness, along with the ability to critically reflect on these aspects. Additionally, they increased their self-confidence, accompanied by an enhanced sense of control and self-efficacy to overcome challenges.

##### Self-understanding

Positive changes in improved understanding of the self were reported by 14.7% (*n* = 7) of iCBT and 15.9% (*n* = 7) of iIPT clients. Enhanced self-awareness and self-understanding are shown by quotes such as *“I understood things about my personality, I understood why certain things bother me and I know how to deal with it now “* (iIPT12) and "*I discovered new things, namely that I was hard on myself*" (iCBT36). The IBIs allowed participants to become more self-compassionate, as illustrated by quotes such as "*I became better to myself and no longer see myself as a bad person*" (iIPT38), and "*I became less harsh on myself*" (iCBT25).

##### Self-efficacy

The participants (12.2%, *n* = 6 iCBT; 18.2%, *n* = 8 iIPT) reported increased self-efficacy and confidence, as reflected in quotes such as “*I control myself more and I can do whatever I think I want to do”* (iIPT32). This growth in self-assurance was further emphasized when participants noted “*I developed self-confidence and self-acceptance*" (iCBT4) and "*I'm no longer afraid and trust myself*" (iIPT35).

##### Positive outlook

This subcategory describes an enduring attitudinal change and captures the shift in how participants view their problems and life situations, reflecting a more optimistic perspective (8.2%, *n* = 4 iCBT; 9.1%, *n* = 4 iIPT). This illustrates how the development of a more positive outlook represents a fundamental change in participants' way of thinking and viewing the world and is described with quotes *such as “The program…gave me hope…and I see positive things for myself despite the circumstances around me*” (iCBT24). This change in outlook was reported by 8.2% (*n* = 4) of the iCBT participants and 9.1% (*n* = 4) of the iIPT participants as quoted here: "*I have had many changes, the first of which is my outlook on the problems in my life. It has changed. I realized that some problems do not exist on the ground as much as they exist within me*" (iIPT19).

##### Depression knowledge

This subcategory emerged as a component of personal growth, where 4.1% (*n* = 2) of iCBT and 2.3% (*n* = 1) of iIPT participants reported developing a deeper understanding of their depressive symptoms. This enhanced understanding represents more than just the acquisition of factual knowledge; it reflects a shift in how participants comprehend their emotional experiences and their condition. The participants demonstrated this growth through statements such as "*I started to understand depression*" (iIPT4) and the ability to differentiate between emotional states: "*I started to know how to distinguish between depression and sadness*" (iCBT7). This improved understanding of their condition contributes to personal growth by enabling participants to develop a more nuanced and informed perspective of their experiences.

#### Positive changes—interpersonal changes

The participants (26.5%, *n* = 13 iCBT; 40.9%, *n* = 18 iIPT) reported experiencing social changes at different levels, such as the quality of relationships or social participation, e.g., leaving isolation and being able to set boundaries in relationships.

##### Social participation

The improvements extended to broader social engagement, with participants actively breaking patterns of isolation (12.2%, *n* = 6 iCBT; 18.2%, *n* = 8 iIPT). One participant expressed this transformation as follows: "*My social relationships improved, I go out more into society, build relationships*" (iCBT5). Others mentioned resuming social activities, such as "*I laugh again and meet my friends*" (iIPT2) and "*reconnecting with previous relationships* " (iIPT14).

##### Quality of relationships

The iCBT (12.2%, *n* = 6) and iIPT (11.4%, *n* = 5) clients reported improvements in their relationship quality with friends, family members or spouses. As one participant noted, "*My relationship with my husband improved and he noticed the calmness, as well as my relationship with my sister*" (iCBT37). The participants described becoming "*more open in relationships*" (iCBT31) and experiencing themselves as more confident in their ability to use communication skills, as quoted here: "*I learned how to be proactive in relationships and communicate*" (iIPT22).

##### Setting boundaries

The participants (4.1%, *n* = 2 iCBT; 11.4%, *n* = 5 iIPT) developed a better understanding of relationship patterns and boundary settings. This included the ability to identify and end harmful relationships, as shown by statements such as "*Breaking off harmful and exploitative relationships—all through therapy*" (iIPT14).Other participants noted the improved ability in "*setting boundaries in relationships with others*" (iCBT17) as well as experiencing themselves as braver, as described here: "*I became braver to speak up*" (iCBT31).

#### Positive changes – increase in activity and energy ('طاقة')

The findings revealed improvements in behavioral activation and energy levels among the participants (26.3%, *n* = 13 iCBT; 20.5%, *n* = 9 iIPT). The improvements manifested across multiple functional domains, encompassing both basic self-care activities (e.g., going for a walk) and more complex goal-directed behaviors (e.g., work). The participants reported renewed engagement in both daily living activities, as expressed in the following quote: "*I started again to clean the house, cook, go outside*" (iIPT24), and productive pursuits "*I have energy—I can work and study*" (iCBT15). The participants generally reported reengaging in activities such as "*I am doing activities that I have not done for a long time*" (iIPT32). This activity pattern is often described via the Arabic term 'طاقة' (energy/strength), meaning increased vitality. The participants specifically documented re-engagement with previously abandoned activities, marking a return to predepression behavioral patterns.

### Negative changes

Most participants actively denied the question (“Has anything changed for the worse for you since therapy started?”) and emphasized that they had grown personally as a result of the treatment, as shown in the following quote: "*There are no negative changes because I have become more aware of my personal spaces, my behavior, and my wrong thinking patterns. Yes, it took me a lot of effort, but I am happy.*" (iCBT21). However, deterioration was mentioned by 11.8% of the clients (10.2% iCBT; 13.6% iIPT). Our analysis identified two distinct categories of negative changes: *short-term negative changes* manifesting during treatment (e.g., treatment-related exhaustion and anger) and more *persistent negative changes* (e.g., hopelessness and emotional numbness; Table 4).

**Table 4:** Negative changes perceived by participants separated by treatment group

Main category	iCBT (*n* = 49)	iIPT (*n* = 44)
Short-term Negative Changes	**6.1% (3)**	**9.1% (4)**
Persistent Negative Changes	**4.1% (2)**	**4.5% (2)**

*Notes:* iCBT = internet-based cognitive behavioral treatment; iIPT = internet-based interpersonal treatment; multiple categories could be coded for each participant. The numbers in bold represent the main categories

#### Short-term negative changes

Treatment-associated short-term worsening of symptoms and negative emotional states while working on the exercises, with participants reporting elevated levels of anxiety and anger, are expressed in the following quotes: *“Just some periods of anxiety during treatment"* (iIPT44); *"When I was writing the letters, I felt provoked and the anger was growing"* (iCBT7). Being confronted with personal and emotional topics during the treatment led to difficulties in tolerating and controlling emotions, as one participant described: “*A lack of emotional control has been causing me to have many negative emotions, especially in the last half of the treatment; negative change: Emotions are triggered by writing*” (iCBT5). Some participants reported feeling exhausted because of the treatment and the exercises. They reported feeling more sadness while writing the letter because their mood deteriorated: "*In a certain exercise, when I used to do alternative thoughts, I felt the opposite, when I used alternative thoughts, my mood was getting worse, not better*" (iIPT25). These effects emerged as direct responses to treatment, suggesting that the treatment process might temporarily heighten sadness, anger or a lack of emotional control.

#### Persistent negative changes

Negative changes that have been reported beyond the treatment period include feelings of hopelessness (*"Sometimes, when I feel there is no hope because there are no solutions"* iIPT7), numbness (*“I became a little dull—I could not cry*” iIPT37), and physical changes (*"My sexual energy"* iIPT2). One person answered the following: “*I am giving myself harsh words and have begun to let depression be the reason I do not do what I should*” (iIPT7).

### Causes of changes

Subjective causes of change were categorized into four main categories: *Program Structure, Personal Factors, Working Alliance,* and *External Resources (*see Table 5*)*. In general, it became clear that participants mentioned more than one cause for the changes and explained them through a combination of different causes. The iCBT participants reported on average *M* = 1.7 different causes, and the iIPT participants reported on average *M* = 1.8 different causes.

**Table 5:** Relative and absolute frequencies of perceived causes of changes separated by treatment group

Main Category	Subcategory	iCBT (*n* = 49)	iIPT (*n* = 44)
**Characteristics of Internet-based Intervention**		**79.5% (39)**	**84.0% (37)**
	Exercises	46.9% (23)	43.1% (19)
	Treatment	22.4% (11)	22.7% (10)
	Written format	10.2% (5)	18.2% (8)
**Personal Factors**		**38.7% (19)**	**36.3% (16)**
	Self-understanding	20.4% (10)	18.2% (8)
	Self-motivation	18.4% (9)	18.2% (8)
**Relationship**		**28.6% (14)**	**25.0% (11)**
	Guidance	22.4% (11)	20.5% (9)
	Opening perspectives	6.1% (3)	4.5% (2)
**External resources**		**16.32% (8)**	**15.9% (7)**
	Social Support	8.1% (4)	11.4% (5)
	Leisure activities, work, and education	8.1% (4)	4.5% (2)

*iCBT* internet-based cognitive behavioral treatment, *iIPT* internet-based interpersonal treatment; multiple categories could be coded for each participant. The numbers in bold represent the main categories

#### Causes of changes – characteristics of the internet-based intervention

Characteristics of the Internet-based Intervention were the category most often mentioned (77.6% (*n* = 38) of iCBT and 84.0% (*n* = 37)). We identified three subthemes for this category: exercise, treatment and written formats.

##### Exercises

The iCBT (46.9%, *n* = 23) and iIPT (43.1%, *n* = 19) participants attributed changes to treatment exercises. Treatment exercises in general were reported as helpful and allowed participants to reflect on different aspects of their lives and address their problems: *"I feel that the exercises have helped to organize my subconscious in a big way"* (iCBT28). The participants described specific methods as helpful, such as dealing with negative thoughts. Working with thoughts seems to have allowed participants to understand the impact that thoughts have on their daily lives and well-being: *"With therapy, I realized to what degree thoughts can affect us and how we deal with thoughts"* (iIPT8). Exercises focusing on resources aimed at directing attention to daily successes and positive experiences and enhancing awareness of positive aspects in clients' lives were also mentioned. The participants reported becoming more grateful through these exercises: *"What helped me most was when I wrote about positive things in my day and became more grateful for things in my life"* (iIPT15; Arabic:), but also to be able to value small achievements during the day: *"The technique of writing especially positive things during the day—also evaluating the day—I felt I was achieving daily even if simple things"* (iCBT7). In addition, analyzing relationships, which involves examining interpersonal dynamics and patterns, helps in understanding and improving relationships and related personal needs (this method was only part of the iIPT treatment): *"What helped me most was thinking about alternatives and possible outcomes and thinking about needs from relationships"* (iIPT32).

##### Treatment

When asked, "In general, what do you think has caused these various changes? ", 22.4% (*n* = 10) of iCBT participants and 22.7% (*n* = 10) of iIPT participants provided responses related to the treatment. That was visible quotes such as “*I benefited from the treatment*” (iCBT34) or “*the treatment helped me to change for the better”* (iCBT47).

##### Written format

Furthermore, 10.2% (*n* = 5) of the iCBT and 18.2% (*n* = 8) of the iIPT participants attributed changes, especially to the written format of the intervention, i.e., relating to the written delivery method of the treatment content, the text-based interactions, and written exercises: *"The therapist's help and the program, everything was useful, especially the writing method, and the direct questions helped me a lot"* (iIPT29). The quotes underline that participants were able to reflect through writing as expressed by one participant: *"The writing, which was like a mirror for me"* (iIPT9) but also to release pressure and tension as quoted in the following: *"The therapist makes me write about topics and I release the charges inside me and come up with solutions by myself"* (iIPT39).

#### Causes of changes – personal factors

Personal Factors as causes of change encompass two key aspects: Self-understanding and Self-motivation. They were mentioned by 36.7% (*n* = 19) of the iCBT participants and 36.3% (*n* = 16) of the iIPT participants.

##### Self-understanding

Self-understanding describes the process of self-discovery and self-comprehension. The participants’ responses revealed that they attributed changes to an improved understanding of themselves: *"I understood myself more"* (iCBT29), which also allowed them to be more gentle to themselves: *"I felt that I am too hard on myself"* (iCBT29). Self-understanding also helped participants be more self-reflective and react more adaptively to symptoms or thoughts: *"I now look at myself when I'm wrong and try to understand the mistake, and this has helped me a lot in my relationships with others"* (iIPT40). This subcategory was mentioned by 20.4% (*n* = 10) of the iCBT participants and 18.2% (*n* = 8) of the iIPT participants.

##### Self-motivation

Self-motivation refers to the clients’ internal drive for change and self-improvement and leads to the hypothesis that successful internet-based therapy also requires intrinsic motivation, as expressed in response to the question “What do you think has caused these various changes?”. This category was reported by 18.4% (*n* = 9) of the iCBT participants and 18.2% (*n* = 8) of the iIPT participants. The own will for change is shown in the following quote: *"I feel I got here alone because I want change and I want to improve myself"* (iCBT4). Similarly, the participants emphasized their intrinsic commitment to therapy as follows: *"I think the reason was my personal desire and will to commit to therapy"* (iIPT27). The participants also acknowledged the support of the program: *"The therapy helped me and I had the determination to help myself"* (iCBT32) or *"Things combined as a team from the therapist and from me too"* (iIPT25The answers show a combination of treatment-related and personal factors that were attributed as a reason for change and underline the importance of successful therapy.

#### Causes of changes – working alliance

A frequently reported cause of change (28.6%, *n* = 14 iCBT; 25%, *n* = 11 iIPT) was the working alliance. The participants reported that counselors were emphatic and actively listened, that they understood by counselors, and that counselors’ explanations during treatment positively changed their understanding and perspective. The three identified subcategories (guidance, perspective opening and empathic understanding) are described in more detail below.

##### Guidance

The participants (22.4%, *n* = 11 iCBT; 20.5%, *n* = 9 iIPT) felt supported by the presence of counselors. Having someone who was there and “listened” whenever needed gave them a feeling of being cared for, which was reported as a cause of change. Clients experienced emotional support and felt recognized, with the therapist providing consistent support and availability, as shown in the following quotes: "*the presence of the therapist and her being there always listening to me helped me a lot*" (iIPT44). It seems that the communication via the program gave participants the feeling that the counselors were always available and that they were not available alone, which was particularly important when dealing with symptoms, as quoted below: "*I feel I got here because …… the therapist's help for me because if I were alone without the therapist, I would not have found any change or improvement*" (iCBT4). Clients felt understood, accepted, and taken seriously by the counselor's empathic responses, as quoted below: *"The therapist made me feel that she understands me very well"* (iIPT4). The visual anonymity of the format and the nonjudgmental and accepting environment of the counselors helped participants be honest, as quoted below: "*The changes were because I was talking to someone I did not know during therapy, so I was very honest—and the therapist did not judge me and guided me, and this is what I needed*" (iCBT20).

##### Perspective opening

This subcategory includes descriptions of counselors helping clients recognize alternative viewpoints. This approach enabled participants to interpret situations more constructively and develop new understandings of their experiences as quoted here: "*The therapist would shed light on things for me to think about, she would give me the thread's end and draw my attention to things I needed to rethink*" (iCBT15*)*. Counselor letters not only encouraged participants to reflect on themselves and reconsider previous interpretations but also facilitated deeper perspectives, as one participant noted: "*The therapist mentioned points that I noticed in myself*" (iIPT34). This subcategory was mentioned by 6.1% (*n* = 3) of the iCBT participants and 4.5% (*n* = 2) of the iIPT participants.

#### Causes of changes—external resources

Among iCBT clients, 16.3% (*n* = 8) and among iIPT clients 15.9% (*n* = 7) attributed changes to external resources such as social support, leisure activities, work and education.

##### Social support

In contrast to generally positive changes, "social support" specifically focuses on the interpersonal level. It describes not only an improvement in the individual condition but also the support received from the social environment. The category was reported by 8.1% (*n* = 4) of the iCBT participants and 11.4% (*n* = 5) of the iIPT participants. Changes were at least partly attributed to social support received from family and friends, as shown in the following quote: *"When I found someone who understands what I'm going through and supports me, like my relative who knew about my situation and took a lot of burden off me"* (iCBT11).Reconnecting with social circles plays a crucial role in participants' well-being, as support from friends might provide a sense of stability and encouragement. This experience is described below: *"My friends' support when I went back to communicate with them"* (iIPT12).

##### Leisure activities, work, and education

This subcategory encompasses beneficial life circumstances that positively impact the therapeutic process. This becomes clear in the following answers to the question “In general, what do you think has caused these various changes?”: “*That I found a job*” (iIPT30), “*I am reading a book currently*” (iCBT24), or *“I also supported myself by entering a program to interpret the Qur'an*” (iCBT35). iCBT (8.1%, *n* = 4) and iIPT (4.5%, *n* = 2) clients reported that subcategory. These external factors play a role in supporting changes alongside internal and therapeutic processes. This is illustrated by the continuation of participant iCBT15’s quote: “*The therapist would shed light on things for me to think about, she would give me the thread's end and draw my attention to things I needed to rethink… and I was listening to podcast*" (iCBT15).

### Hindering aspects

In terms of hindering treatment aspects (asked for the question “Which parts/aspects about internet-based therapy have been hindering for you?”), three main thematic categories were identified (Table 6): Characteristics of Internet-based Interventions (46.9%, *n* = 23 iCBT; 45.5%, *n* = 20 iIPT), Lack of Tailoring (8.1%, *n* = 4 iCBT and 18.1%, *n* = 8 iIPT), *Technical Difficulties* (12.3%, *n* = 6 iCBT; 2.3%, *n* = 1 iIPT) and External Reasons (2.1%, *n* = 1 iCBT and 2.3%, *n* = 1 iIPT). Approximately one-third of the participants reported no hindering aspects at all (32.7%, *n* = 16 iCBT; 38.6%, *n* = 17 iIPT).

**Table 6:** Relative and absolute frequencies of hindering parts separated by treatment group

Main Category	Subcategory	iCBT (49)	iIPT(44)
**Nothing**		**32.7% (16)**	**38.6% (17)**
**Characteristics of Internet-based Interventions**		**46.9% (23)**	**45.5% (20)**
	Written Format	26.5% (13)	13.6% (6)
	Exercises	8.2% (4)	24.9% (11)
	Response time & Duration	12.3% (6)	6.8% (3)
**Lack of Tailoring**		**8.1% (4)**	**18.1% (8)**
	Topics	0.0% (0)	15.9% (7)
	Lack of Personalization	8.2% (4)	2.3% (1)
**Technical Difficulties**		**12.3% (6)**	**2.3% (1)**
**External Reasons**		**2.1% (1)**	**2.3% (1)**

*iCBT* internet-based cognitive behavioral treatment, *iIPT* internet-based interpersonal treatment; multiple categories could be coded for each participant. The numbers in bold represent the main categories

#### Hindering aspects – characteristics of internet-based intervention

Characteristics of the internet-based Intervention were the most frequently mentioned category of hindering aspects reported by 46.9% (*n* = 23) of the iCBT participants and 45.5% (*n* = 20) of the iIPT participants. We identified three subthemes for that category: written format, exercises and response time and duration.

##### Written format

The Written Format was described as hindering or difficult, and a preference for “verbal parts” was reported by 26.5% (*n* = 13) of iCBT and 13.6% (*n* = 6) of iIPT participants as quoted here: *"The written therapy was good and helped me, but I would have preferred it if there* was a part of a call*"* (iCBT21). The participants reported difficulties in expressing themselves in a written way: *"Sometimes I could not write at the same time I had trouble writing my feelings"* (iIPT7) as well as being afraid of being misunderstood, as stated in the following: *"Barriers are the difficulty of expressing written based on the therapist because I am afraid of not being understood"* (iCBT14). By reporting a preference for phone calls, it became clear that participants were also open to synchronous live communication, as quoted in the following: *"if there was a phone conversation between sessions, it would be better"* (iIPT4).

##### Exercises

The participants described the treatment exercises as hindering because of the specific content (8.2%, *n* = 4 iCBT; 13.6%, *n* = 11 iIPT) or framework, such as instructions and length. Daily exercises (e.g., thinking about conflict-related situations with others) were described as hindering, as participants reported that they did not fit their situation: *"Sometimes I could not find situations and it is not a condition that every day something happens"* (iIPT36). Exercises were also described as too long or too difficult, and instructions were perceived as being unclear: *"Sometimes there are assignments that are difficult to understand or do not resemble situations I have experienced, and I do not know what to write in them"* (iIPT42). Furthermore, participants reported that the exercises were an additional burden in their daily life: *"Writing the assignment and I did not have enough time because my day was busy"* (iIPT28).

##### Response time and duration

Counselors were instructed to respond to the clients’ letters within 48 h. The waiting period was perceived as too long by some clients, prompting a reported preference for traditional sessions that offer more immediate access, as reflected in the following quote: “*Replying after two days, I would have preferred an open conversation with a time limit at the same time”*. (iIPT25). Treatment duration in general was described as too short: *"I wish the program had a longer duration"* (iCBT49). Those themes were reported by 12.3% (*n* = 6) of iCBT and 6.8% (*n* = 3) of iIPT clients.

#### Hindering aspects – lack of tailoring

In this theme, statements were categorized that indicate the standardized structure of treatment as obstructive. Two subthemes were identified: Aspects that focus on the topics addressed by the program and aspects that convey a lack of individualization.

##### Topics

Hindering aspects regarding the topics of the sessions were mentioned only by participants of the iIPT condition (15.9%, *n* = 7), who reported a wish to talk about other topics and did not understand why relationships and their problems were discussed in such detail during treatment: *"What was bothering me was that the therapy was focused only on relationships and I needed to talk about other things… I had no problems in relationships so this was making writing difficult"* (iIPT3)). Because they did not have problems in relationships, *"Some of the letters did not concern me like relationships because I had no problems in relationships in general"* (iIPT5).

##### Lack of personalization

Some participants (8.2%, *n* = 4 iCBT; 2.3%, *n* = 1 iIPT) reported a lack of personalized response letters from their counselors: *"I had thoughts that the messages were not meant for me"* (iCBT31), and connected that to the counselors’ interest: *"There were points that she [the therapist] did not answer as if she was tired and did not want to hear anything else"* (iIPT41).

#### Hindering aspects—technical difficulties

Hindering aspects were also mentioned related to the platform itself (12.3%, *n* = 6 iCBT; 2.3%, *n* = 1 iIPT), as it was described as slow: *"The program is very slow and gets stuck, especially on the messages page"* (iCBT22), and complicated: *"At the beginning, I was distracted and did not understand how to access the messages and had difficulty navigating the program itself"* (iCBT28), which made the treatment exhausting: *"Writing on the website so I had to manually write the letters and then transfer them to the program, which was exhausting"* (iCBT20). The participants mentioned that notifications did not arrive on time: *"Delayed delivery of messages to my personal email address they arrived late and I needed to login by my own to see the message"* (iCBT4).

#### Hindering aspects—external reasons

Two participants (2.1%, *n* = 1 iCBT; 2.3%, *n* = 1 iIPT) reported not having much time for the treatment because of life circumstances such as work: *"I did not have enough time to write the assignment because I had a busy day and the writing task was exhausting"* (iIPT28), or studying: *"It’s something personally, because I was in a period of study and it was difficult with studies and I needed to be dedicated"* (iCBT37.

### Difficult but helpful

The participants were asked to report aspects of the treatment that were difficult but helpful. The iCBT clients (32.7%, *n* = 16) and iIPT clients (34.1%, *n* = 15) reported that the treatment was not difficult and were therefore unable to recall difficult aspects. Other answers were related only to the difficult part of the question:

One person was not able to describe what exactly was perceived as difficult (“*There are difficult aspects, but I don’t know how to describe them*”; iCBT30). The participants (4.3%, *n* = 2 iCBT; 2.3%, *n* = 1 iIPT) perceived the questionnaires as exhausting, repetitive and difficult: “*The difficulty lies in the set of questionnaires, which takes hours to complete.”* (iIPT34). Other difficulties related to the topics and the start of the intervention in general (4.3%, *n* = 2 iCBT; 9.1%, *n* = 4 iIPT). All other reported aspects related to the question “What was difficult but helpful” were classified under the *Characteristics of Internet-based Interventions* category (see Table 7).

**Table 7:** Relative and absolute frequencies of difficult but helpful aspects separated by treatment group

Main Category	Attribution	iCBT (49)	iIPT(44)
**Characteristics of Internet-based Interventions**		**55.1% (27)**	**56.8% (25)**
	Written Format	16.3% (8)	11.4% (5)
	Exercises	40.8% (20)	45.5% (20)

*iCBT* internet-based cognitive behavioral treatment, *iIPT* internet-based interpersonal treatment; multiple categories could be coded for each participant. The numbers in bold represent the main categories

#### Characteristics of internet-based interventions

More than half of the reported difficult but helpful aspects are related to the structure of the Internet-based Intervention (55.1%, *n* = 27 iCBT; 56.8%, *n* = 25 iIPT) including the written format and the exercises.

##### Written format

The Written Format (16.3%, *n* = 8 iCBT; 11.4%, *n* = 5 iIPT) was described as challenging, time-consuming, and demanding, yet beneficial: *"The writing process was time-intensive and challenging; however, it proved beneficial"* (iIPT27). In particular, the beginning of the writing process was perceived as difficult but proved helpful: *“I was feeling heavy to write my thoughts, but the therapist encouraged me and I was pushing myself and the writing experience was very useful*” (iIPT29).

##### Exercises

Treatment-related exercises (40.8%, *n* = 20 iCBT; 45.5%, *n* = 20 iIPT) were reported as challenging but beneficial. Exercises requiring participants to recall specific situations, engage in reflection or daily exercises were characterized: *"The process of recalling and documenting specific situations presented expressive challenges; nevertheless, it facilitated my ability to overcome them"* (iIPT5). The writing exercises were perceived as challenging, demanding, and time intensive, while simultaneously beneficial: *"Certain exercises proved challenging and fatiguing—particularly the alternative ideas exercise, which, despite its difficulty, demonstrated significant utility"* (iCBT20).

## Discussion

This study provides valuable insights into how Arabic-speaking individuals experience IBIs, illuminating their perceived changes (beneficial and adverse), and their subjective evaluation of helpful and hindering aspects of the treatments. Furthermore, the analysis provides insights into two distinct therapeutic approaches (iCBT and iIPT) and the participants' experiences across these approaches. This understanding is particularly important, as, to the best of our knowledge, the present study is one of the first qualitative investigations specifically examining the perspectives on the IBIs of Arabic-speaking participants, most of whom are living in Arabic-speaking countries. Thus, it contributes not only to the understanding of how therapeutic approaches may be experienced across different populations but also to the vision of possible improvements from a client’s perspective.

Both iCBT and iIPT participants reported positive changes not only in symptom improvement but also across multiple domains, such as coping strategies, personal growth, interpersonal changes, and increased activity and energy levels. For iCBT participants, the three most frequently reported positive change categories were *Coping Strategies, Personal Growth*, and *Symptomatic Changes*. For iIPT participants, the most commonly reported categories for positive change were *Personal Growth, Interpersonal Changes,* and *Coping Strategies.* Lindegaard et al. [35] identified five overarching themes among Arabic-speaking participants in Sweden, including "the importance of being seen" and "new ways of knowing and doing". These categories align with our identified categories. The participants reported an enhanced understanding of their symptoms, their impact, and management strategies, alongside increased self-awareness and reflection. Although *Symptom Changes* were reported by clients from both conditions, it was the third most frequently mentioned category in the iCBT condition. Except for *Symptom Changes*, those identified domains are not measured in the seven scales commonly used to capture improvement by patients with depression in research [21]. This finding underlines the existing debate in clinical care away from an exclusive focus on symptom reduction toward a more comprehensive understanding of recovery. The discrepancy between primary clinical outcomes in controlled trials and the patient perspective highlights the need to consider multidimensional outcome domains such as psychosocial functioning, quality of life, participation, and personal growth [12].

The high frequency of reported *Coping Strategies* across both iCBT and iIPT conditions emphasizes the significance of this outcome in IBIs, regardless of the therapeutic approach used. The participants in the iCBT condition frequently reported the effective use of cognitive coping, in which they learned to address difficult situations and identify both negative and positive aspects. The fact that this category was mentioned by more than 50% of iCBT participants underpins that this core component (i.e., cognitive restructuring) of the approach was targeted effectively by the IBI. In the iIPT condition, Interpersonal Changes were identified as the second most frequently reported category, suggesting that the treatment's primary focus on interpersonal relationships was also targeted effectively. However, a substantial subset of iIPT participants reported difficulties in understanding the relationship-focused approach (e.g. as they perceived no difficulties in their relationships) and would have preferred to address other topics. While some participants benefited from the strong focus on relationships, others could not understand why this was the focus of the treatment. This underlines the negative side effects of the standardization of the study protocol, as participants were randomly assigned to either the iCBT or iIPT treatment groups. A challenge that might be addressed by the personalization of treatment [13, 14]. Asking about individual participants’ preferences and needs, such as "Do you experience difficulties in your relationships?" before the treatment rather than the randomization approach used in this study might improve the fit with the treatment.

*Personal Growth* was often reported across both treatment conditions and appeared to be the most frequently mentioned category for iIPT clients. The participants emphasized enhanced self-awareness and increased self-efficacy after treatment. The participants reported developing greater self-understanding, specifically recognizing patterns of self-criticism, and learning to adopt a more empathetic stance toward themselves. Although self-understanding was not a primary treatment target, several modules incorporated elements that promoted this attitude (e.g., writing a letter to oneself). The participants' reports of increased self-understanding suggest that the interventions were effective in addressing this factor even without explicit emphasis and underlined a positive side effect. This finding is particularly important given recent meta-analytic evidence confirming the negative relationship between self-compassion and depression [25].

The majority of participants reported no negative changes. However, adverse effects were reported by a total of 11.8% of the participants and were categorized into two main domains: *short-term* and *persistent negative changes*. The proportion of participants reporting at least one negative change in the study was comparable to the findings of Rozental et al. [46], in which an average of 9.3% of IBI participants reported at least one adverse event. Importantly, our methodology involved the systematic categorization of all participant responses indicating any adverse outcomes (e.g., hopelessness, numbness, or physical changes). Current literature lacks a standardized operational definition of negative therapeutic change. Future research would benefit from a more rigorous conceptualization and empirical investigation of this construct to enhance methodological precision and clinical relevance [47]. The identified categories align with the Rozental et al.'s [46] classification of treatment-related and patient-related negative effects. This underlines the importance of considering adverse effects during but also after treatment, for example, using checklists for adverse effects and monitoring them during and after treatment (e.g., [48]). However, further empirical research in this area is needed to elucidate the underlying mechanisms and to identify reliable predictors of adverse therapeutic outcomes to enable timely clinical intervention and harm reduction strategies.

The participants predominantly attributed causes of changes to structural elements of the *Internet-based Intervention*, including the overall treatment framework, written format, and therapeutic exercises, with only minimal differences descriptively observed between iCBT and iIPT. Notably, the participants of the study conceptualized writing as a reflective tool, characterizing it as a "mirror" that enhanced self-understanding. The perceived value of the written format aligns with findings from Patel et al.'s [44] qualitative review, which revealed that written expression facilitated uninterrupted self-reflection and emotional articulation. Personal Factors (self-motivation and self-understanding) emerged as the second most frequently reported cause of change across both groups. Treatment success was consistently associated with participants' intrinsic motivation and desire for improvement. This finding also corresponds with Patel et al.'s [44] identification of initial motivation, encompassing hope, and self-management as crucial determinants in IBIs for depression. The attribution of change to personal agencies appears particularly salient in IBIs, potentially facilitated by the inherent flexibility and autonomy these platforms offer (e.g., deciding when and where to work on the therapeutic exercises). Furthermore, IBIs were found to foster participant empowerment, enabling the development of coping strategies and enhancing self-efficacy. This facilitates a transition from passive to active engagement in condition management. These findings are in line with results from systematic reviews of the literature highlighting significant positive effects of IBIs on empowerment, self-efficacy and mastery compared with waiting list, usual care, or no care [6, 50]. The third most common category in terms of attribution of change was the *Working Alliance*. The participants characterized their counselor as empathetic and understanding, particularly valuing that the counselor "listened" and provided support. Notably, participants used verbal communication terms such as "listen" and “talk”, although the intervention was written and asynchronous, suggesting that participants experienced a sense of being *heard* in written format. However, this topic needs more research in the future. The qualitative review of Patel et al. [44] identified 18 studies in which a therapeutic relationship was built remotely. Their observation underlines our findings that therapist‒patient relationships are characterized as collaborative partnerships. The current results underline the importance of guidance in the context of IBIs. Compared with unguided interventions, guided internet-based interventions for depression have larger effect sizes, greater acceptability, higher levels of engagement with the intervention platform, and lower attrition rates [10, 27, 58]. The fourth category encompassed *External Resources*, including social support networks and recreational activities. While perceived social support was identified as a cause of change as well as perceived positive change, it has not been reported by other qualitative studies from client perspectives [35, 52]. Patel et al. [44] reported that social support was predominantly reported in group interventions rather than in traditional or internet-based interventions. However, the association between perceived social support and mental health is well established through quantitative research [1, 51, 60]. Gaines et al. [22] demonstrated that both iCBT and iIPT achieve comparable changes in perceived social support, which aligns with our findings of similar participant reports across conditions. The significance of social support may be particularly pronounced in Arabic-speaking countries, where family and social networks play a central role [23]. While Lindegaard et al. [35] interviewed Arabic-speaking individuals in Sweden, notably, the participants were forced to leave their countries and were likely disconnected from their original social networks. This interpretation is supported by Böge et al.'s [7] findings, which demonstrate differential support patterns of refugee groups. Other support was significantly correlated with reduced depressive symptoms among refugees in Germany, whereas family support was significantly associated with reduced PTSD symptoms among refugees in Jordan. Our findings underscore the importance of incorporating external factors, such as social support and meaningful activities, into depression treatment protocols. The results emphasize the multifaceted nature of therapeutic change in IBIs and highlight the interplay between program structure, personal agency, therapeutic alliances, and environmental support systems.

Interestingly, while most participants attributed positive changes to Characteristics of the Internet-based Intervention, a substantial number also described structural elements as hindering factors, and some described them as difficult but helpful. This supports the category “Treatment format not for everyone” identified by Lindegaard et al. [35]. In particular, the written format was also described as hindering by some participants, aligning with previous findings on IBIs [32, 35, 44, 53]. This highlights the importance of treatment personalization and a good fit between patients' preferences and the treatment approach. In this context, a need for direct communication with therapists was also expressed, either through verbal exchanges or real-time chats. Some participants suggested that such interactions do not need to be weekly but could be incorporated periodically throughout the treatment process. Others perceived the written form as difficult because they did not receive an immediate response to their letters. These findings indicate that the internet-based treatment approach may not be universally suitable but might be suitable for specific groups. While IBIs are low threshold and independent of time and place, they might create other barriers, such as literacy, language skills, attention span, and, in the case of writing interventions, written-based emotional expressive ability. In addition, specific aspects, such as the 48-h counselor response time, were perceived as too long and hindering. The participants in the iCBT group also reported technical challenges, including platform performance and delayed reminder notifications, which should be considered when an intervention is implemented. These observations align with insights from iCBT therapists, who recognized the limitations of digital-only treatment formats. They emphasized that some patients benefit from in-person interactions and that such programs may not be suitable for everyone [9]. Although the therapeutic exercises were generally well received in our study, some participants perceived them as being too long, complex, and exhausting. These findings reinforce the notion that standardized solutions may not be optimal for all participants, suggesting the need for more individualized, need-based approaches to treatment delivery and high demand for patients with depressive symptoms [9].

### Limitations

This study offered access to an underserved population, characterized predominantly by educated, urban, female participants from Arabic-speaking countries (primarily Egypt). However, the results need to be interpreted with consideration of the following limitations. First, only treatment completers who agreed to participate in interviews were included, creating a double selection that likely overrepresents positive treatment experiences. This becomes clear when one considers that people who participated in the interview tend to have fewer symptoms after treatment than non-interviewed participants. Non-completers and interview decliners may have had systematically different experiences that remain unexplored. Second, interviews were conducted retrospectively at the end of treatment rather than during active treatment, introducing recall bias and limiting insights into session-by-session experiences and real-time treatment processes. This timing prevented the capture of evolving perceptions and immediate reactions to interventions. Third, the structured interview guide, while ensuring standardization across multiple interviewers, may have restricted the discovery of unexpected themes that more inductive, open-ended approaches could have revealed. Additionally, real-time note-taking rather than audio recording likely reduced data depth, missing nuanced responses, nonverbal cues, and precise participant language. While suitable for the current research context, future studies with different objectives could benefit from more open, in-depth interviews. Furthermore, the analysis lacked subgroup differentiation. No typological analysis was performed to identify distinct patient groups (e.g., those reporting exclusively positive, negative, or mixed experiences), which could have provided more nuanced insights into treatment heterogeneity. A further limitation concerns the validation of the intervention content. While the iCBT and iIPT programs were adapted by licensed psychotherapists and based on Interapy, an empirically validated internet-based treatment protocol [33, 49], a formal expert panel review to assess content validity was not conducted prior to implementation. Although the efficacy trial demonstrated significant symptom reduction in both active treatment conditions compared to waitlist control [16], future implementations of culturally adapted IBIs should incorporate structured expert review processes to ensure both cultural appropriateness and therapeutic accuracy of intervention content. However, our results may not generalize to individuals who did not complete treatment or declined participation, who may have had systematically different experiences. The predominantly educated, urban, and female composition of our sample limits applicability to populations with different sociodemographic characteristics. Furthermore, while participants came from various Arabic-speaking countries, the majority were from Egypt; findings may not equally apply to other cultural contexts within the Arabic-speaking countries. Finally, experiences within this research trial context, with structured therapist feedback and defined treatment protocols, may differ from other IBI implementations.

## Conclusion

The internet-based CBT and IPT approaches for depression are subjectively experienced as effective by Arabic-speaking participants. These findings should be interpreted within the boundaries of our sample, which consisted of treatment completers who were predominantly educated, female, and from Egypt; transferability to other populations and implementation contexts requires further investigation. The findings indicate that participants experienced improvements not only in depressive symptoms but also across multiple domains, including coping strategies, personal growth, interpersonal changes, and activity levels. This suggests that IBIs facilitate comprehensive psychological change beyond symptom reduction and illustrates that improvement should be considered multidimensionally, not solely through symptom measures in psychotherapy research. However, individual differences in experience are notable, as different aspects were described both as helpful and hindering by different participants. The findings suggest that a less standardized approach might improve the acceptance of IBIs even more and that patients might benefit from more personalization and tailoring, which might be addressed with interim assessments guiding the treatment approach, adaptations that might also be implemented by adding intermediate questions, among others. The findings also indicate the value of incorporating participants' subjective experiences more extensively in evaluation processes to better understand individual treatment trajectories. Specifically, for clinicians, these findings highlight the importance of monitoring not only symptom change but also broader domains such as coping, personal growth, and interpersonal functioning. Clinicians should remain attentive to individual differences in how participants experience structured interventions. For IBI developers, the dual role of program structure suggests that incorporating adaptive features, such as flexible pacing, optional modules, or intermediate assessments guiding treatment intensity, may improve acceptability and engagement.

## Supplementary Information

Supplementary Material 1. Table S1. Coding Matrix. Table S2. Original Interview Quotes.

## Data Availability

The data generated and analyzed during this study are not publicly available due to their sensitive nature. The transcripts and field notes contain detailed personal narratives, mental health experiences, and contextual information that could potentially compromise participant anonymity, even with standard de-identification procedures. This restriction is consistent with the ethical approval granted by the Freie Universität Berlin Ethics Committee and reflects the informed consent agreements signed by all participants, which guaranteed confidentiality and specified that raw data would not be shared beyond the immediate research team. Researchers interested in accessing the dataset for scientific purposes and meet the criteria for access to confidential data, may submit a formal request to the research department of Center Überleben gGmbH (contact via mail@ueberleben.org) for consideration under appropriate data sharing agreements.

## Abbreviations

CBT: Cognitive Behavioral Therapy
GAD-7: Generalized Anxiety Disorder-7 scale
iCBT: Internet-based Cognitive Behavioral Therapy
iIPT: Internet-based Interpersonal Psychotherapy
IPT: Interpersonal Psychotherapy
M: Mean
MSPSS: Multidimensional Scale of Perceived Social Support
PHQ-9: Patient Health Questionnaire-9
QoL: Quality of Life
SCID-5-CV: Structured Clinical Interview for DSM-5 Disorders
SD: Standard Deviation
