# Supplementary material for: Client perspectives of internet-based treatment for depression in Arabic-speaking countries
Source: BMC Psychol. 2026 Apr 13;14:511. doi: 10.1186/s40359-026-04463-z (PMC13072602; doi:10.1186/s40359-026-04463-z)
Supplement: Supplementary file 1 — Supplementary Material 1. Table S1. Coding Matrix. Table S2. Original Interview Quotes. [file 40359_2026_4463_MOESM1_ESM.docx]

Supplement 1

*Table S1*

Coding Matrix

| **Maincategory** | **Subcategory** | **Definition** | **Example** |
| --- | --- | --- | --- |
| **Perceived changes** | | | |
| **Symptomatic Changes** |  | Direct alterations in core depressive symptomatology and baseline mood state (reduction in dejection, improved overall mood, better sleep quality, energy levels, appetite) | “Fewer depressive episodes”; “Symptoms are much improved, both sleep and appetite” |
| **Coping Strategies** |  | Adaptive strategies utilized by clients to manage depressive symptoms and challenging situations |  |
|  | Cognitive coping | Restructuring negative thoughts, cognitive awareness | “I can turn negative situations and thoughts into positive ones” |
|  | Behavioral coping | Concrete behavioural strategies to deal with problems | “I learned that if I get upset, I write down the things that upset me.” |
|  | Emotion regulation | Active control and regulation of emotions | „I started to control my emotions” |
| **Personal Growth** |  | The process of developing a better understanding of oneself, building confidence in one’s abilities, gaining knowledge about personal challenges, and fostering a more positive and hopeful outlook on life. |  |
|  | Self-understanding | New insights about the self, own patterns and needs; recognising self-criticism/excessive demands; understanding own limits and values | “I understand myself better” |
|  | Self-efficacy | Increased confidence in own abilities; feeling of control and ability to act | “I started to be able to control my life now” |
|  | Positive outlook | Hopeful outlook for the future |  |
|  | Depression knowledge | Knowledge about the disorder | „I now know the difference between depression and sadness” |
| **Interpersonal Changes** |  | Transformations in the relational domain of clients' lives; It includes the process of emerging from social withdrawal and engaging in communal activities, qualitative improvements in relationships, and the development of healthy boundary-setting capacities within interpersonal contexts |  |
|  | Social participation | Leaving isolation and participating in social activities | “I started to interact with friends again” |
|  | Quality of relationships | Qualitative changes in relationships | „My relationship to my husband improved” |
|  | Setting boundaries | Development of boundary setting in relationship | “I cut toxic relationships” |
| **Increase in Activity and Energy** |  | General increase in activity level, resumption or resumption of activities | “I've been doing activities in my day” |
| **Perceived causes of changes** | | | |
| **Program Structure** |  | Reported aspects related to the treatment structure itself (e.g. writing, exercises, duration) and perceived as cause for reported changes |  |
|  | Exercises | Attribution to treatment related excercises | “The exercises during the therapy” |
|  | Treatment | Attribution to the internet-based treatment | “The treatment in general” |
|  | Written format | Attribution to the writing process | “The written letters” |
| **Personal Factors** |  |  |  |
|  | Self-understanding | Attribution to the process of self-understanding | “Because I learned more about myself” |
|  | Self-motivation | Attribution to own motivation to change | “ I wanted to change” |
| **Working Alliance** |  |  |  |
|  | Guidance | Attribution to the therapist's support; feeling of being heard, feeling of acceptance, feeling taken seriously, feeling that is there and is listening | “The therapist was always there and listend”; “The therapist understood me” |
|  | Opening Perspectives | Therapist opens up new perspectives, helps to see/classify things differently | “The therapist made me think” |
| **External Resources** |  |  |  |
|  | Social Support | Attribution to external social support, active improvement of social relationships | “ My contact to my friends became more”; “My family supported me” |
|  | Leisure activities, work, and education | Attribution to leisure activities, external educational resources, work | “I started a Koran course” |
| **Perceived hindering aspects** | | | |
| **Characteristics of Internet-based Interventions** |  | Reported hindering aspects related to the treatment structure itself (e.g. writing, exercises, duration) |  |
|  | Written format | Writing process is described as hindering/difficult | “Sometimes I needed to talk to some to be able express myself” |
|  | Exercises | Treatment exercises described as hindering/difficult |  |
|  | Response time & Duration | Response time from therapist is described as too long and treatment is described as too short | “I have to wait two days to get a respond, that was long for me” |
| **Lack of Tailoring** |  |  |  |
|  | Topics | Treatment topics are perceived as inappropriate or not understandable because they do not apply to the person | “I did not understand why I have to talk about my relationships, I don’t have problems” |
|  | Lack of Personalization | Perceived absence of individualized letters | “Sometimes I felt the letters were not meant for me” |
| **Technical Difficulties** |  | Difficulties while engaging with the digital intervention platform; related to program performance speed, interface complexity and navigation obstacles, delayed system notifications, and problems with message retention or saving functionality | “Reminder messages arrived too late” |
| **External Reasons** |  | Difficulties because of personal life circumstances like daily tasks | “I was to busy with work and life” |
| **Difficult but helpful** | | | |
| **Characteristics of Internet-based Interventions** |  | Aspects related to the treatment structure itself (e.g. writing, exercises, duration) and perceived as difficult but helpful |  |
| **Written Format** |  | The writing process is described as difficult but helpful | "The writing itself took time and wasn't easy, but it was rewarding.” |
| **Excercises** |  | Exercises are described as difficult but helpful | “There were exercises that were difficult and I got tired of them - such as the alternative ideas exercise, which was very difficult even though it was useful.” |

Supplement 2

*Table S2*

Original Interview Quotes

| ID | Quote |
| --- | --- |
|  | *Positive Changes* |
| iCBT16 | لاحظت تغيير نحو الأفضل أتحول من إنسان كئيب إلى سعيد برجع *للوضع الطبيعي الآن* |
| iCBT1 | *نوبات الاكتئاب أصبحت أقل في شدة الأعراض* |
| iIPT44 | *الأعراض الجسدية من ألم الظهر و ألم بالمعدة تحسنت كثيرا* |
| iIPT9 | كنت كتومة ومكتئبة وحزينة وضعف طاقة وكل ده راح كل شيء تحسن الأفكار السلبية كلها تغيرت |
| iCBT47 | *افكر فى المواقف و تحليلها و البحث عن السلبيات و الايجابيات* |
| iCBT27 | *صرت أبحث بهاد الموقف السلبي على أي إشي إيجابي لحتى يغطيه* |
| iCBT44 | *الأفكار السلبية صرت أتحكم عليها* |
| iIPT29 | *بدأت أفكر قبل أن أقوم بشيء، و لاأنصاع وراء أفكاري السلبية و كيف أضع بدائل للأمور* |
| iCBT11 | تعلمت إذا تضايقت أنو أكتب إيش الأشياء يلي مضايقتني وكمان المواقف السلبية أحاول أحلها وصار عندي تغيير في أفكاري وسلوكي وصرت واعية أنه لما أكون منزعجة أعمل تمارين تنفس أطلع أمشي أعمل اشي بيومي |
| iIPT36 | *بقيت أسيطر على مشاعري شويه* |
| iIPT25 | *صرت أعبر أكثر عن مشاعر بلا تراكما* |
| iCBT24 | *قلت انفعالاتي مو مثل قبل* |
| iIPT12 | فهمت حاجات في شخصيتي فهمت ليه حاجات معينة بتضايقني وهعرف اتعامل معاها بعد كده |
| iCBT36 | ا*كتشفت أمور جديدة وهي أني قاسية على نفسي* |
| iIPT38 | *أنا بقيت أحس بنفسي أكتر اني ما بقتش أشوف نفسي شخص وحش* |
| iCBT25 | *بقيت أقل قسوة على نفسي* |
| iIPT32 | بتحكم في حالي أكتر والشيء اللي بفكر أني بدي اعمله بقدر اعمله |
| iCBT4 | *صار عندي ثقة ورضى عن ذاتي* |
| iIPT35 | *مبقتش خايفة وواثقة من نفسي* |
| iCBT25 | *البرنامج ...عطاني امل ....وأشوف أشياء إيجابية بنفسي على الرغم من الظروف اللي حولي* |
| iIPT19 | *صار عندي تغيرات ملانة أولها هي وجهة نظري بالمشاكل يلي بحياتي تغيرت أدركت أنه* *بعض المشاكل مش موجودة على أرض الواقع بقدر ما هي موجودة بداخلي* |
| iIPT4 | أ*اصبحت أفهم ما هو الإكتئاب*) |
| iCBT7 | *أصبحت أعرف أميز بين الإكتئاب والحزن* |
| iCBT5 | *علاقاتي الاجتماعية تحسنت صرت أطلع للمجتمع صار عندي علاقات* |
| iIPT2 | *اصبحت أضحك و أقابل أصحابي* |
| iIPT14 | *إعادة تواصل مع علاقات سابقة* |
| iCBT37 | *تحسنت علاقتي بزوجي وهو لاحظ الهدوء وكذلك علاقتي بأختي* |
| iCBT31 | *صرت منفتحة أكثر في علاقاتي* |
| iIPT22 | *عرفت كيف أكون مبادرة في العلاقات والتواص*ل |
| iIPT14 | *قطع العلاقات المؤذية و الاستغلالية من حياتي - كل ذلك من خلال العلاج* |
| iCBT17 | *تحديد الحدود في العلاقات مع الآخرين* |
| iCBT31 | *صرت أكثر شجاعة أني أتكلم* |
| iIPT24 | *بقيت أرجع أرتب البيت أطبخ أخرج* |
| iCBT15 | *صار عندي طاقة - بقدر أشتغل وأدرس* |
| iIPT32 | *بعمل نشاطات ما كنت بعملها بقالي زمان* |
|  | *Negative Changes* |
| iCBT21 | *تغييرات سلبية لا ما في لأنه صرت مدرك لمساحاتي الشخصية أكثر لسلوكي لأنماط تفكيري الخاطئة هذا صح أخذ مني مجهود كثير بس أنا مبسوط* |
|  |  |
| iIPT44 | *فقط بعض فترات من القلق أثناء العلاج* |
| iCBT7 | *عندما كنت أكتب الرسائل كنت أشعر بالاستفزاز وأن الزعل بيزيد* |
| iCBT5 | عدم التحكم في المشاعر بقت بتجيلي مشاعر سلبية كتير في مرحلة خاصة في النصف الأخير من العلاج; التغيير السلبي : خروج المشاعر بسبب الكتابة |
| iIPT25 | *في تمرين معين كنت لما باجي اعمله الأفكار البديلة كنت بحس بالعكس لما بحط أفكار بديلة كانت حالتي المزاجية بتسوء مش تتحسن* |
| iIPT7 | *أحياناً لما أشعر أن مفيش أمل لأن مفيش حلول* |
| iIPT37 | بقيت متبلد شوية - مش قادر أعيط |
| iIPT2 | *طاقتي الجنسية* |
| iIPT7 | *أوجه كلام قاسي لحالي أصبحت أخلي الاكتئاب سبب أني ماعملش اللي علي* |
|  | *Causes of changes* |
| iCBT28 | *أشعر أن التمارين ساعدت على ترتيب لاوعيي بشكل كبير* |
| iIPT8 | *مع العلاج استوعبت إلى أي درجة الأفكار ممكن تأثر علينا وطريقة تعاملنا مع الأفكار* |
| iIPT15 | *أكتر شي ساعدني لما كنت بكتب عن الأشياء الإيجابية في يومي وصرت أمتن أكتر للأشياء بحياتي* |
| iCBT7 | *تكنيك التدوين خاصة الأمور الإيجابية خلال اليوم - كذلك تقييم اليوم حسيت بأني بنجز بيومي حتى لو أشياء بسيطة* |
| iIPT32 | أ*اكتر شي ساعدني هو التفكير في البدائل والنتائج المحتملة والتفكير في الإحتياجات من العلاقات* |
| iCBT34 | الإستفادة من العلاج |
| iCBT47 | العلاج ساعدني للتغييرات للأفضل |
| iIPT29 | *مساعدة المعالجة لي و البرنامج كل شيء كان مفيد و طريقة الكتابة خصوصاً، و كانت الاسئلة المباشرة تساعدني كثيراً* |
| iIPT9 | *الكتابة ، التي كانت كالمرآة بالنسبة لي* |
| iIPT39 | *المعالجة بتخيلني اكتب في مواضيع وبفرغ الشحنات اللي جوايا وبطلع حلول بنفسي* |
| iCBT29 | *فهمت نفسي اكتر* |
| iCBT29 | *بدأت أشعر أنني قاسي على نفسي* |
| iIPT40 | *أصبحت أنظر لنفسي عندما أكون* *مخطئة وأحاول فهم هذا الخطأ وهذا ساعدني كثيراً بعلاقاتي مع الأخرين* |
| iCBT4 | *بحس أنا وصلت لحالي هون لأنو بدي التغيير وبدي أحسن من نفسي* |
| iIPT27 | *أعتقد أن السبب كان رغبتى الشخصية و إرادتي فى الإلتزام فى العلاج* |
| iCBT32 | *العلاج ساعدني و أنا كان لدي العزيمة على مساعدة نفسي* |
| iIPT25 | *أشياء مجتمعة كفريق من المعالجة و مني أنا كمان* |
| iIPT44 | *وجود المعالجة و كونها موجودة هناك تسمعني دائماً ساعدني جداً* |
| iCBT4 | *بحس أنا وصلت لحالي هون لأنو ..... مساعدة المعالج ل الي لأنه لو كنت لحالي بدون المعالج ما لاقيت أي تغيير وأي تحسن* |
| iIPT4 | *المعالجة أحسستني بأنها تفهمني جداً* |
| iCBT20 | *التغييرات كانت بسبب أني كنت أحكي مع أحد لا أعرفه أثناء العلاج فكنت صريحة جداً - والمعالجة لم تحكم علي وكانت توجهني وهذا ما كنت أحتاجه* |
| iCBT15 | *المعالجة كانت بتضوي عالأشياء حتى أفكر فيها كانت تعطيني طرف الخيط وتلفت نظري إلى أمور أحتاج أن أعيد التفكير فيها* |
| iIPT34 | *المعالج اعطى لي نقاط لاحظتها في نفسي* |
| iCBT11 | *لما لقيت حدا يفهم ايش أنا بمر وداعم ل الي مثل قريبتي يلي عرفت بوضعي وكثير صارت شايلة حمل عني* |
| iIPT12 | *دعم الصحاب لما رجعت أتواصل معاهم* |
| iIPT30 | *وأنني لقيت عمل* |
| iCBT24 | *أقرأ حاليًا كتاب* |
| iCBT35 | *كمان أنا دعمت حالي أني دخلت برنامج لتفسير القرآن* |
| iCBT15 | *المعالجة كانت بتضوي عالأشياء حتى أفكر فيها كانت تعطيني طرف الخيط وتلفت نظري إلى أمور احتاج أن أعيد التفكير فيها .... وكنت أسمع بودكاست* |
|  | *Hindering Aspects* |
| iCBT21 | *العلاج الكتابي منيح وساعدني بس كنت بفضل يكون في جزء مكالمة* |
| iIPT7 | *أحياناً مكنتش أقدر أكتب في نفس الوقت كان عندي صعوبة أكتب مشاعري* |
| iCBT14 | *لمعيقات هي صعوبة التعبير للمعالج كتابياً الخوف من عدم فهم* |
| iIPT4 | *لو كان بين كل كم جلسة محادثة هاتفية كان أفضل* |
| iIPT36 | *ساعات مش بلاقي مواقف ومش شرط يحصل حاجة كل يوم* |
| iIPT42 | *أحياناً كان هناك مهام صعبة الفهم أو لا تشابه مواقف مررت بها فلا أعلم ماذا أكتب فهيا* |
| iIPT28 | *كتابة المهمة ومكنش عندي وقت كافي لأن يومي مشغول* |
| iIPT25 | الرد بعد يومين كنت أفضل محادثة مفتوحة لها وقت محدد فى نفس الوقت |
| iCBT49 | *كنت أتمنى يكون البرنامج مدته أطول* |
| iIPT3 | *اللي كان بيعيقني أن العلاج مركز فقط على العلاقات وأنا كنت محتاج أتكلم عن حاجات تانية أن ماكنش عندي مشاكل في علاقاتي فده كان بيصعب الكتابة* |
| iIPT5 | *بعض الرسائل لم تكن تعنيني كالعلاقات لأنه لم يكن يوجد لدي مشكلة في العلاقات بشكل عام* |
| iCBT31 | *أفكار لدي أن الرسائل ليست مخصصة لي* |
| iIPT41 | *في نقاط مجوبتنيش عليها كانها بتعبي فراغ ولا تريد سماع أي كلام آخر* |
| iCBT22 | الموقع *بطيء جداً و بيعلق خاصةً فى صفحة الرسائل* |
| iCBT28 | *في البداية كنت متشتت ولم أفهم كيفية الوصول للرسائل وكنت أجد صعوبة في التعامل مع الموقع نفسه* |
| iCBT20 | *الكتابة على الموقع لذا كنت أكتب الرسائل يدوياً ثم انقلها على الموقع وهذا كان متعباً* |
| iCBT4 | *تأخر الرسائل في الوصول لإيميلي الشخصي كانوا يوصلوني متأخرات لازم أفوت أفقد لحتى أشوف الرسائل* |
| iIPT28 | *كتابه المهمة ومكنش عندي وقت كافي لأن يومي مشغول والمهمة كتابية كانت مرهقة* |
| iCBT37 | *هى حاجه خاصة ، لأني كنت فى فترة دراسة و كان صعب مع الدراسة و يحتاج تفرغ* |
|  | Difficult but helpful |
| iCBT30 | هو في حاجات صعبة بس مش عارف أوصفها |
| iIPT34 | الصعوبة فى مجموعة إستبيانات بتاخد وقت ساعات بمل |
| iIPT27 | *الكتابة نفسها كانت تأخذ وقتاً و لم تكن سهلة و لكنها كانت مفيدة* |
| iIPT29 | كنت أشعر في الثقل لكتابة أفكاري لكن المعالجة شجعتني و كنت أضغط على نفسي و كانت تجربة الكتابة مفيدة جداً، |
| iIPT15 | *مثلاً ذكر بعض المواقف التفكير و الكتابة عنها كان بها صعوبة للتعبير عنها لكنها ساعدتني على تجاوزها* |
| iCBT20 | *كان هناك تمارين صعبة وتعبت منها - مثل تمرين الأفكار البديلة كان جداً صعب رغم أنه كان مفيد* |
